# The acceptability and feasibility of a mobile phone delivered brief intervention for hazardous drinking in India

**DOI:** 10.1093/oodh/oqae045

**Published:** 2024-10-29

**Authors:** Abhijit Nadkarni, Danielle Fernandes, Richard Velleman, Anastasia Onyango, Seema Sambari, Ethel D’Souza

**Affiliations:** Centre for Global Mental Health, Department of Population Health, London School of Hygiene and Tropical Medicine, Keppel Street, London, WC1E 7HT, United Kingdom; Addictions and Related Research Group, Sangath, Porvorim, Goa, 403501, India; Addictions and Related Research Group, Sangath, Porvorim, Goa, 403501, India; Addictions and Related Research Group, Sangath, Porvorim, Goa, 403501, India; Department of Psychology, University of Bath, Bath, BA2 7AY, United Kingdom; Addictions and Related Research Group, Sangath, Porvorim, Goa, 403501, India; Department of Sociology, Harvard University, Boston, MA 02138, United States; Addictions and Related Research Group, Sangath, Porvorim, Goa, 403501, India; Addictions and Related Research Group, Sangath, Porvorim, Goa, 403501, India

**Keywords:** complex intervention development, Mhealth, brief interventions, hazardous drinking, India, mixed methods

## Abstract

The effectiveness of Brief Interventions (BIs) for hazardous drinking is well established. However, the implementation of BIs at scale in low- and middle-income countries such as India is rare, primarily due to human resource related barriers. This paper describes the testing of acceptability and feasibility, and the preliminary impact, of a mobile phone delivered BI in an uncontrolled treatment cohort and nested qualitative study. Consenting adult (≥18 years) participants with an Alcohol Use Disorder Identification Test score between 8 to 15 were recruited from educational institutions, workplaces and primary care settings. The TLFB (Time-Line Follow Back) was administered to participants at baseline and 3 months post recruitment. All participants received the BI through text messages or IVR (Interactive Voice Response) over eight weeks; and in-depth interviews were conducted with some participants. The mean pre and post outcomes were compared using paired t-test. Qualitative data was analysed using thematic analyses. 29 participants received the intervention and 16 (55%) completed the outcome assessments. Some key qualitative findings indicated the desire from participants for fewer messages and messages that did not require interaction; and more messages with motivational content and information on practical skills to reduce drinking. The percentage days abstinent was significantly higher at follow up than baseline in all those who had completed the TLFB at baseline and follow up. Feasibility and acceptability testing is an important component of the intervention development process to ensure that the resulting intervention is suitable for the context.

## INTRODUCTION

Alcohol consumption in low- and middle-income countries (LMICs) across the world is steadily increasing in comparison to high income countries (HICs) [1]. India, one such LMIC, is also experiencing an increase in alcohol consumption, as well as alcohol-related problems such as hazardous drinking [2, 3]. Hazardous drinking is a pattern of drinking that puts the drinker at risk of adverse health and social consequences; while harmful and dependent drinking occurs when the alcohol consumption has already started causing social, physical and psychological harms. Compared to the latter two, the prevalence of hazardous drinking is higher in India [4, 5]; however, health policy in the country focuses predominantly on these latter two [6].

There is substantial evidence to support the cost-effectiveness of brief interventions (BIs) in reducing hazardous drinking [7, 8]. BIs are structured intervention strategies of short duration that aim to help an individual to cease or reduce their drinking [9]. BIs generally incorporate some or all of the following elements: feedback on the person’s alcohol use and any alcohol-related harm; clarification as to what constitutes low-risk alcohol consumption; information on the harms associated with risky alcohol use; benefits of reducing alcohol intake; advice on how to reduce alcohol intake; motivational enhancement; analysis of high-risk situations for drinking and coping strategies; and the development of a personalized plan to reduce alcohol consumption [10].

BIs, most commonly informed by Motivational Interviewing (MI) techniques, and delivered by non-specialists (e.g. nurse, layperson) or digitally demonstrate evidence of efficacy in changing both short- and long-term alcohol-related outcomes. The positive outcomes have been demonstrated in males within healthcare settings, university students in primary care settings, helpline-delivered BIs, lay counsellor–delivered BIs and computerized BIs [11]. In general medical settings, alcohol-targeted BIs produce beneficial changes equivalent to a reduction in one drinking day per month [12]. The limited evidence from LMICs indicate short-term gains, single-session BIs fare better than multiple sessions, nurse delivered BIs showing better results than BIs delivered by others, and similar outcomes in young adults and middle-age individuals [8].

Despite evidence of cost effectiveness [7] and apparent simplicity of content, implementation of BIs in LMICs is rare [13]. Barriers to implementation of BIs in routine care include shortage of human resources, high existing clinical workload, competing priorities, perceived complexity of the intervention, as well as social norms related to alcohol use [13].

Technological innovation is one way of overcoming some of these barriers to increased coverage of evidence-based interventions, and there is already some evidence about the utility of technology-based interventions for a range of substance use problems [14, 15]. This is a particularly appealing option in LMICs, such as India, where a ‘mobile-first’-based approach to communications is allowing digital platforms to provide new possibilities for delivering a range of interventions [16]. However, exploration of the potential of using mobile phones as platforms for delivery of BIs must be coupled with more culturally sensitive research, as not much is known about contextual influences on BIs, such as cross-cultural variability, and health system idiosyncrasies, or how existing evidence, primarily from HICs, may be generalized to other healthcare settings [17, 18].

AMBIT (Alcohol use disorders Mobile based Brief Intervention Treatment) aimed to do that by developing and testing a BI for hazardous drinkers, which is contextually relevant and delivered using a mobile phone interface. The specific objectives of AMBIT were to: a) Use a systematic methodology to develop and refine a BI package, informed by global evidence, and modified to be delivered using mobile phone technology; b) Examine the feasibility of delivery and acceptability of the BI delivered using mobile phone technology in a LMIC context; c) Evaluate the preliminary impact of the BI on drinking outcomes; and d) Fine-tune procedures for a definitive Randomized Controlled Trial (RCT) of the BI. This paper describes b) and c) above—the testing of acceptability and feasibility, and the preliminary impact, of the mobile phone delivered BI, which was built through a systematic intervention development methodology [19]. Through a mixed methods intervention development process involving review of global evidence, in-depth interviews with various stakeholders, Delphi survey with experts, and content development workshops with a range of stakeholders we developed an intervention with messages related to safe drinking/health education, alcohol reduction, drinking and risk management, drinking alternatives, situational content, urge management and maintenance and relapse prevention.

## MATERIAL AND METHODS

### Settings

Goa is a small state in Western India with a population of 1.4 million people. Alcohol is easily available and less expensive in Goa as compared to other states in India. Goa has low abstinence rates and high rates of problematic alcohol consumption, reflected in a high burden of alcohol used disorders (AUDs) in primary care, workplaces and young people in educational institutions [5, 20, 21]. Adolescent drinking onset has shown a significantly increasing trend over time from 20% for those born between 1956 and 1960 to 74% for those born between 1981 and 1985 [22]. In community residing male adults (18–49 years) men, over a 6-year period 4% of baseline non-drinkers and 15% of casual drinkers developed AUD, and 47% of hazardous drinkers and 55% of harmful drinkers continued to have AUD [23]. Participants were recruited from two educational institutions (one urban and one rural), five workplaces (a hotel, a brewery, a newspaper office and two manufacturing industries) and two primary care facilities.

### Study design

Uncontrolled treatment cohort with before and after design and nested qualitative study. The study included iterative feedback loops which allowed revision of the intervention based on ongoing analysis of the incoming data. Data obtained through this process was used to iteratively refine the intervention, delivery mechanism, and recruitment procedures, to inform the near-final treatment package to be tested in a pilot RCT. Participant recruitment was conducted from December 2018 to August 2019, and the outcome assessments and qualitative interviews were conducted from February to November 2019.

### Sample

Participants were identified using the Alcohol Use Disorder Identification Test (AUDIT) a 10-item screening questionnaire for AUD, developed by the WHO [24]. The AUDIT has been validated and used in cross-national studies, including in India [25] and has been field-tested in the study setting [26]. Eligibility criteria included adult (≥18 years) males with an AUDIT score between 8 to 15 (hazardous drinkers), and in possession of a personal mobile phone. Although the overall prevalence of drinking among women in India is low, there is emerging evidence about the increasing prevalence of alcohol use in young women in educational institutions [5]. Hence, in the educational institutions and primary care settings, females aged 18–25 years, who fulfilled the other criteria described above were also recruited. In recognition of stigma around seeking treatment for problematic alcohol consumption, low participation rates were anticipated in the educational institutions and workplaces. Hence, to increase the acceptability of the screening in those settings, ‘Health and Wellness Camps’ were conducted where the screening questions about alcohol use were combined with the International Physical Activity Questionnaire (IPAQ) [27], and Perceived Stress Scale (PSS) [28].

### Baseline data

The following data was collected: socio-demographic information, preferences for receiving the intervention (e.g. preferred language to receive it). Intervention preferences were shared with our technology partner to ensure customized delivery of the intervention. The TLFB (Time-Line Follow Back) method was used as a baseline measure of alcohol consumption. It is a calendar method of collecting data about quantity and frequency of drinking and various memory aids are used to enhance recall. The TLFB, which has high test–retest reliability and concurrent validity has been established in various types of AUD, and has been previously used at the study site [29].

### Intervention

Each participant received either SMS (Short Message Service or text) messages or IVR (Interactive Voice Response) calls on their mobile phone over eight weeks. The average character count for each message was ~150. Each week’s messages were focused on specific content areas derived from the formative research phase of the study [19]. Seven themes guided the content of the weekly messages: Safe drinking/health education, alcohol reduction, drinking and risk management, drinking alternatives, situational content, urge management and maintenance and relapse prevention. The last theme guides the last 2 weeks of intervention delivery, while the rest guide the messages for one week each. The messages were a mix of push (not requiring a response from the recipient) and pull (requiring a response from the recipient) messages. Everyone received the push messages (e.g. ‘self-awareness’) while only some received the pull messages based on the information they provided (e.g. ‘actionable feedback’ for urge management). Once the participants consented to receive the intervention, they received the intervention content through regularly scheduled messages. Once the messages were delivered, the participants could access the content as and when they would like. The content of the IVR messages was exactly the same as the SMS messages.

The proposed active components of the intervention were building awareness through information, enhancing self-awareness through reflection on one’s drinking in relation to the information received and subsequently setting drinking goals, actionable feedback through evidence-based strategies for reducing problematic drinking behavior, enhancing motivation, goal monitoring through regular check ins, and skills to maintain change and prevent relapse. Further details of the intervention content are available in Appendix S1. During the period of intervention delivery, the data manager and program coordinator monitored the delivery of the intervention for each participant and addressed any challenges faced.

### Outcomes

At three months post-recruitment, the TLFB was administered to each participant. The TLFB was then used to calculate the primary outcome measure of frequency of drinking, Percent Days Abstinent (PDA); secondary outcomes of quantity of drinking, standard drinks consumed in past 2 weeks; and pattern of drinking, Percent Days Heavy Drinking (PDHD).

In addition, in-depth interviews (IDIs) were conducted with participants—some immediately after the intervention delivery and some after the 3-month outcome evaluation. The IDIs were conducted with all 16 participants who completed the outcome evaluation and with two who did not. The interview questions were informed by the research objectives of the study and sought to collect data on perspectives and feedback on the content areas and delivery mechanisms of the intervention. The interview guide aimed to understand the participant’s experiences of receiving the intervention, e.g. accounts of challenges faced, opinions of delivery styles that were conducive to participation, feedback on the usefulness of different content areas and perceived impact on their alcohol use.

### Ethics

AMBIT was approved by the host institution’s Institutional Review Board and the Indian Council for Medical Research. All harmful or dependent drinkers who were identified, received a BI in the form of an information leaflet and were also given information about services provided by trained counsellors at the host institution. Anyone identified with high stress or low physical activity received an informational leaflet with guidance to improve physical and mental wellbeing.

### Analyses

The quantitative data was analysed using STATA SE 14. Descriptive analyses were conducted using t-test and chi square tests for means and proportions respectively. The mean median pre and post outcomes were compared using paired t tests the Wilcoxon matched-pairs signed-rank test, a nonparametric method to compare before-after or matched subjects. The qualitative data was analysed by two independent coders (DF, SS) using Nvivo version 11. The IDIs were first translated (wherever appropriate) and transcribed. The transcripts were then analysed using a thematic analysis approach. The analysis involved generation of codes from raw data, followed by deriving themes by retrieving pieces of data pertaining to codes and examining their meaning in relation to the research questions (acceptability of the content and delivery of the mobile phone-based intervention, barriers to engagement, perceived impact on drinking habits).

## RESULTS

**Figure 1:**
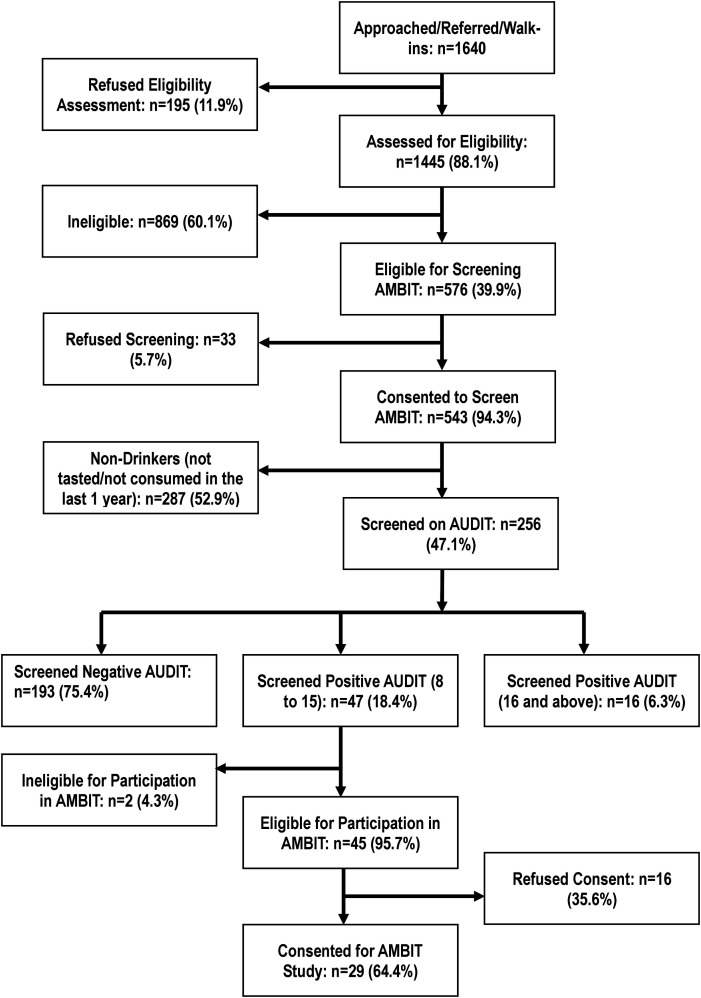
Recruitment flowchart.

Participants were recruited to treatment cohort through a sequential process (Fig. 1). We gained access to 1640 participants from educational institutions, workplaces and primary care facilities, through universal screening or referrals. Of these, 1445 (88.1%) agreed to be assessed for eligibility for screening, and 576 of them (39.9%) were eligible for screening. The main reasons for refusal to be assessed for eligibility included ‘not having time’ (43.1%), ‘not interested’ in the study (13.3%) and concern about missing their position in the queue to see the doctor in the clinic (37.9%). The main reasons for ineligibility for screening were as follows: didn’t meet the age criterion [18–65 years] (12.3%), female [in the workplace settings] (64.6%), already screened for AMBIT (11.5%), not residing in the catchment area for the entire duration of the study (8.1%), difficulty with hearing or speaking (1.6%) and others (1.7%). Only three (0.3%) were ineligible because they did not own a phone.

Of those who consented for screening (n = 543), 287 (52.9%) were never drinkers or not current drinkers (not consumed any alcohol in past 12 months). The rest (n = 256) were screened using the AUDIT. Of these, 47 (18.4%) were hazardous drinkers, 16 (6.3%) were harmful drinkers (AUDIT score > 15), and the rest had no AUD. Two were excluded as they were not conversant with responding to SMS or IVR, and 45 met the criteria for inclusion in the case series. Of these, 29 (64.4%) consented to participate and were enrolled in the case series. Two of these participants opted for IVR and the rest opted for SMS intervention. There were no statistically significant differences between those who consented and those who did not, on socio-demographic indicators and baseline AUDIT score. The reasons for refusal included ‘not interested in the study’ (50%n = 8), ‘no time’ (25% n = 4) could not understand the concept of the study (12.5%n = 2) and other reasons (12.5%n = 2).

The participants included 15 students from educational institutions (two females), eight males from workplaces and six patients (one female) in primary care. Table 1 summarizes the characteristics of the study participants.

**Table 1:** Characteristics of study participants.

**Variable**	**Quantitative** **N = 29** **N (%)**	**Qualitative** **N = 18** **N (%)**
**Gender**		
Female	3 (10.3)	1 (5.6)
Male	26 (89.7)	17 (94.4)
**Mean age (SD), Range**	27.7 (12.2), 18–55	28.8 (13.2)
**Marital status**		
Never married, divorced, widowed	21 (72.4)	13 (72.2)
Married	8 (27.6)	5 (27.8)
**Education**		
Completed secondary school	4 (13.8)	1 (5.6)
Completed higher secondary school	23 (79.3)	16 (88.9)
Graduate	2 (6.9)	1 (5.6)
**Employment status**		
Employed	12 (41.4)	6 (33.3)
Unemployed	1 (3.5)	0 (0)
Student	16 (55.2)	12 (66.7)
**Mean AUDIT score (SD)**	11.1 (2.1)	10.8 (2.1)
**Recruitment site**		
Primary care	6 (20.7)	3 (16.7)
Workplace	8 (27.6)	4 (11.2)
Educational institution	15 (51.7)	11 (61.1)

The participants received the mobile phone-based intervention over an 8-week duration, and the outcome assessment was conducted three months post-recruitment. All 29 participants completed the intervention (completion was defined as receiving the intervention package for the two-month duration), and 16 (55%) participants completed the quantitative outcome assessment. Reasons for drop out included participants a) reporting not having time to complete outcome evaluation (n = 1), b) reportedly not being interested in outcome evaluation (n = 5), c) not responding to phone calls to fix outcome evaluation appointment (n = 6), d) relocating and not being contactable (n = 1). Except for gender, there were no statistically significant differences on sociodemographic indicators and baseline AUDIT score between those who completed outcome evaluation and those who dropped out (Table 2).

**Table 2:** Comparison of participants who completed outcomes assessments and those who were lost to follow up.

**Variable**	**Completed outcome assessment** **N = 16 (55.2%)** **N (%)**	**Lost to follow up** **N = 13 (44.8%)** **N (%)**	**p**
**Gender**			0.04
Female	0 (0)	3 (100)	
Male	16 (61.5)	10 (38.5)	
**Mean age (SD)**	29.8 (13.7)	25.2 (10.0)	0.32
**Marital status**			0.62
Never married, divorced, widowed	11 (52.4)	10 (47.6)	
Married	5 (62.5)	3 (37.5)	
**Education**			0.41
Completed secondary school	1 (25.0)	3 (75.0)	
Completed higher secondary school	14 (60.9)	9 (39.1)	
Graduate	1 (50.0)	1 (50.0)	
**Employment status**			0.43
Employed	6 (50.0)	6 (50.0)	
Unemployed	0 (0)	1 (100)	
Student	10 (62.5)	6 (37.5)	
**Mean AUDIT score (SD)**	11.0 (2.0)	11.2 (2.1)	0.77
**Recruitment site**			0.84
Primary care	3 (50.0)	3 (50.0)	
Workplace	4 (50.0)	4 (50.0)	
Educational institution	9 (60.0)	6 (40.0)	

Table 3 describes the difference in the three outcomes between baseline and follow up. One participant reported visiting his native village in the period covered by the TLFB at follow up. During this period, he drank heavily every day and he specified that this was different from his usual pattern. As a result, we undertook an analysis after excluding this outlier. The PDA was statistically significantly higher at follow up than baseline in all those who had completed the TLFB at baseline and follow up. For the other two outcomes there were no statistically significant differences between baseline and follow up. After including the outlier, the change in PDA remained statistically significant, and for the other two outcomes the change was not statistically significant (Appendix S2).

**Table 3:** Difference in drinking patterns at baseline and follow up.

**Outcome**		**All participants excluding outlier (n = 15)**	
		**Median (Range)**	**p value**
Percent Days Abstinent (PDA)	Pre-treatment Post treatment	57.1 (0.0–92.9) 92.9 (71.4–100.0)	0.0001
Percent Days Heavy Drinking (PDHD)	Pre-treatment Post treatment	0 (0.0–28.6) 0 (0.0–28.6)	0.31
Standard drinks	Pre-treatment Post treatment	8.8 (0.0–51.0) 3.1 (0.0–39.3)	0.11

The majority of the participants who participated in the IDIs were from educational institutions, one was a female, majority were single and had completed at least higher secondary schooling (Table 1).

Their feedback was used to refine the content areas and delivery mechanisms of the intervention package. The results are organized under three major themes—content, delivery and outcomes.

**A) Content**

1. Information

The intervention package included a significant amount of information on the consequences of hazardous drinking, as well as the benefits to be expected from managing alcohol use. This information was largely appreciated by the participants. Several spoke about the importance of knowing the daily/weekly limit of consumption beyond which the drinking is considered risky.

*“First you change their* (drinker’s) *minds. People these days believe in more facts. You'll say, ‘you plan to take 6 drinks this week. Alright! That will have x alcoholic percentage and taking more than x percentage will be bad. So, you should drink within this amount’. That will act as a trigger, for them to say ‘Okay, now I need to control’.”* —Female, 21 y

2. Behavioural strategies

While discussing the content covered in the intervention, some participants stated a preference for content that focused on strategies for non-risky drinking, rather than abstinence. Some participants also requested more guidance on managing challenges related to reducing alcohol use, particularly peer pressure.

*‘Yes, that is why I liked the messages, and I was reading. Because when we go to the doctor, the doctor says if you drink, then this will happen to you. But in your messages, you don't tell us to stop, neither do you ask us to drink.’—*Female, 21 y

*‘But at least I feel that the ones responsible to turn the youngsters to drinking are their friends, nobody else. How to save yourself from these friends, I want a solution for this. If four people are sitting to drink and I am sitting with them, I cannot leave. This solution should be given. There is self-control but there is also peer pressure. How can that be changed?’*—Male, 18 y

3. Motivating anecdotes

Several participants also appreciated content that motivated them to manage their drinking, such as personal stories. Some also requested for motivational quotes to be incorporated.

*“Stories as well, people telling their own stories, could help a lot. If I know someone is affected due to drinking or drugs, I might stop drinking after seeing him or listening to him.” —*Male, 21 y

*“Including some quotes which help like, "you should stop doing it." "It’s never too late." "Better late than never," If you send some quotes like that, it will make it beneficial.”* —Male, 20 y

4. Multimedia content

Some participants suggested the use of multimedia, such as videos and images, to deliver information. This option was explored in detail by the research team but was not adopted due to limitations with the SMS/IVR delivery platform.

*‘If you send a video or something animated, it will be good. You will get a positive response. It will be effective because whatever we visualize, we understand. For weak learners, reading becomes a challenge. So instead of reading, it is better to watch the 3 minutes video and visualize it.’—*Male, 18 y

**B) Delivery**

1. Amount of content

When discussing engagement barriers, many participants expressed the time constraints they faced. Similarly, many mentioned that the length and number of messages acts as a deterrent to engagement and stated a preference for shorter messages and fewer number of messages in a day. Furthermore, some suggested alternate days as the ideal frequency for sending messages.

*“I actually wanted to respond but I got caught up with some work. So, I think I interacted 3-4 times. There were messages saying, ‘let's play a game’. But I was busy, and I could not attend to them.” —*Male, 20 y

*“When we are at work, it is not possible to read the messages. If it is very lengthy, will we work or read the message? And there is no guarantee that the full message will be read. We would just focus on the main points. If it is in short, then it will be better.” —*Male, 47 y

2. Interactive ‘push and pull’ component

The preliminary intervention package had a highly interactive format. This included a ‘push and pull’ format; where participants were required to reply to messages and based on these responses, personalized feedback would be delivered. However, participants enrolled in the case series described a lack of desire to respond to the messages and preferred to simply receive the intervention content.

*“Some messages asked to, ‘press 1 to enter’ or ‘press 1 to know more’. That sometimes becomes irritating for teenagers and other people who do not like texting. If we get 2-3 messages and some information, that is fine. But for something like this, we get bored, and we don't want to reply anymore. Sometimes we get* (other) *notifications, we get busy and switch to those. Now who will reply to so many messages? That is the only drawback”* —Female, 21 y

*“I feel that sending only information will be enough. I think there is no need to respond. Why should one respond? When one reads, everything is understood.” —*Male, 48 y

3. Customised delivery timings

Some participants described occasions when they considered the intervention messages inapplicable to them, such as days when they were not engaging in alcohol use or periods of religious significance that require abstinence. Messages enquiring about current alcohol use during these occasions were considered irrelevant, and this acted as an engagement barrier, precluding the participant from responding. Participants also suggested customizing delivery timings to days of heightened alcohol use (e.g. cultural events, parties).

*“If they ask, how much did you drink today? But I have not drank anything, so I don’t have an answer. So, one message of the day will fail.”* —Male, 18 y

*“Maybe you can update according to the parties or events happening in your area. If there is an event happening on Thursday night, you can text on that day.” —*Female, 21 y

4. Delivery platform

The participants largely found the mobile phone-based platform acceptable for intervention delivery. The easy accessibility of the platform, as well as its time and cost efficiency were cited as merits. On the other hand, some of the participants, particularly the older participants, also described the benefits of face-to-face interventions.

*‘I liked this better than going to the doctor. Because we can receive this anywhere, like in the house or in the office.’—*Male, 35 y

*‘If a person goes for counselling, he pays for the counselling. He will have to spare the time and take his appointment every day. But the college students are getting this intervention, it's a very good job. Thanks for this.’—*Male, 18 y

*‘Now the way you are sitting and talking to me* (talking to the researcher undertaking the outcome interview)*, it feels peaceful. If this happens every day, then it will have an effect. This is my opinion.’—*Male, 50 y

While discussing the mobile phone-based platform, several participants suggested the use of social messaging applications (e.g. WhatsApp, Instagram, Facebook Messenger) as an alternative to SMS/IVR. Messaging applications were described as platforms that are already widely used and hence, more convenient. On the other hand, SMS/IVR platforms are less used and messages or calls from this platform are often mistaken for advertisements or spam. Participants also preferred these platforms due to their ability to support multimedia content, which was deemed more attractive and engaging.

*‘Now SMS messages come only from companies. Because everybody has a smartphone and uses social media. Instagram, WhatsApp, Facebook, or Twitter. So, youngsters interact more on this, as compared to SMS messages.’—*Male, 18 y

*‘People do not have much concentration for text messages. When receiving it on WhatsApp, if there is a picture or a video and there is an explanation given, then people can read it and they will grasp it.’—*Male, 42 y

**C) Perceived outcomes and mechanisms of change**

1. Drinking patterns

Most participants described a reduction in alcohol use, which included decreased consumption as well as a decrease in frequency. Some participants also described a change in drinking patterns within their social group, because of dissemination of the intervention content to their peers.

*“It’s been more than a month since I drank. I was drinking a bottle a day. Then I came to half. Then I stopped completely. So, it was a good experience after quitting.” —*Male, 21 y

*“Earlier we would plan to bring bottles and have a drink. Now we plan to drink slowly. We enjoy the night, or we enjoy the whole day with just one bottle. When I stopped, they also did. I used to forward the messages to them.” —*Male*,* 21 y

While speaking about their decrease in alcohol consumption, participants also described changes in behaviors associated with their drinking routines. This included employing safe-drinking strategies, and techniques to regulate consumption.

*“After drinking, we used to go to a friend's room or go out with the group to drink. Now we have decided to take a person who doesn't drink, just to be on the safer side. He drives the car. When I had drinks, I stayed there along with my friend. This happened fifteen days ago. I drank and I stayed in his room. I said I don't want to ride back home. Because I knew that to drink and drive is not good.” —*Male, 42 y

2. Reasons for change

When discussing the reasons for reductions in their alcohol use, participants spoke about the influence of the intervention acting in different ways. Some described the motivation from the intervention as an important facilitator of change, where simply receiving a message/call acted as a reminder.

*“I liked that there was somebody to guide. Nowadays you find less people to guide you to good things. And you find more of those who tell you bad things. So, when I found this, I felt better.” —*Male, 47 y

Others spoke about the influence of increased awareness about their own drinking and the possible adverse outcomes. This included learning about the health consequences of heavy alcohol use, as well as awareness of their own drinking patterns. For some, hearing that their alcohol consumption is considered hazardous acted as an impetus to manage their drinking.

*“Earlier I was consuming a bit too much alcohol and had pressure from family and friends that I should not drink. That it's bad for health. So, I was thinking that I should probably stop drinking. When the researcher interviewed me for the first time* (at screening)*, I thought "Okay this is the best opportunity". The messages and what she told helped me.” —*Male, 20 y

Finally, some participants also spoke about their responsibilities towards family or friends and their recognition of the negative effect of excessive alcohol use in this respect.

*“I did not go to drink. I thought about how much loss this causes. You lose your money, also it affects your diet, your health will get affected. I have my family; my children are young. Why to simply drink? Now I have read information about this.” —*Male, 47 y

### Iterative feedback

The feedback indicated the desire for some specific cognitive (motivational content) and behavioral (skills to navigate social environments that promote/encourage drinking). Accordingly, we added content to the messages to ensure that these crucial domains were adequately addressed. Examples of such messages included the following: ‘*Have a healthy meal before you drink, sip your drink slowly and space out an alcoholic drink with snacks, water and non-alcoholic drinks.*’ (Behavioral content). ‘*As you start a healthy lifestyle, reflect on these words by Dr Abdul Kalam. “You cannot change your future, but you can change your habits, and surely your habits will change your future.*”’ (Motivational content).

We also received consistent feedback about the frequency and delivery timings of the messages. When we started off, the pre-determined frequency of the messages based on the formative research was 5–7 messages per day over 2 days a week. However, the feedback from participants indicated that this was not desirable, and in fact interfered with engagement. Hence, we revised that to 3–4 messages per day over 3 days a week. Additionally, there were suggestions to customize delivery timings to days of heightened alcohol use. Such a modification would have required more active engagement from the participants (akin to Ecological Momentary Assessment) [30], and our evidence indicated that participants wanted less interactive engagement, not more. Hence, after much consideration we decided that such a level of customization would not be feasible for a scalable intervention.

One key characteristic of our preliminary intervention, informed by our formative research, was its interactive nature. However, during intervention delivery we observed that the engagement with such a ‘push and pull’ format was very low (the response rate to such messages was only 27.8%). This was further enforced by the data from the IDIs, which indicated that participants did not particularly want such an intense engagement with the intervention. Hence, we modified the intervention framework from a predominantly interactive ‘push and pull’ format (17 interactive messages), which necessitated responses from the participants, with a predominantly ‘push’ format (only nine interactive messages), where messages are sent without necessitating a response. This resulted in an increase in the response rate to 37.3% for the interactive messages.

Finally, based on the data from the IDIs we extensively explored the possibility of delivering the intervention through messaging platforms. We examined several potential options such as Facebook Messenger and WhatsApp. However, we ultimately decided to continue with SMS for reasons of scalability (ease of access in rural areas compared to other messaging platforms which require data packages), concerns around data privacy and limitations of automated delivery of messages for those platforms. However, as such platforms become more accessible and amenable to secure use for healthcare delivery, they could become preferred options as they are more suitable for more engaging multimedia content.

## DISCUSSION

This paper presents the acceptability, feasibility and preliminary impact testing of a mobile phone delivered BI for hazardous drinking that we developed using a systematic intervention development methodology [19]. This involved i) testing the acceptability and feasibility of procedures to identify and recruit participants in a range of settings, ii) iteratively refining the treatment package through participant feedback, and iii) testing the feasibility, acceptability and preliminary impact of the intervention.

Process data from the study indicated that it is feasible to identify and recruit patients with hazardous drinking from a range of settings. While universal screening is a more efficient system in primary care where the prevalence of hazardous drinking is higher than in the general population, screening in non-healthcare settings required a much more holistic process addressing other wellness issues that resonate with the general population. A combination of these systems resulted a very high level of acceptability of screening and reasonably high consent rate to receive an intervention for something that they had not been seeking help for in the first instance i.e. hazardous drinking.

Two other key observations were about ownership of mobile phone and choice of receiving the messages, important considerations for feasibility and acceptability of our intervention. An extremely small proportion of participants were ineligible because they did not own a phone. Furthermore, among those who consented to participate, only a couple opted to receive the intervention over IVR. These were important observations for separate reasons. Although the high, and increasing, tele-density in India is a potential opportunity for mHealth initiatives, there are still concerns about the reach and coverage reliability of telecom networks, and the proficiency/comfort of mobile phone owners with the various functionalities of their phones [31]. Our findings indicate that these are not key concerns, at least for the settings in which we implemented our study. One area of concern around the feasibility of study procedures is the moderately high dropout from outcome assessment. This is relatively high as compared to some of our previous work involving face to face counselling, in which follow up rates have been higher than 80% [26]. However, this could be due to the nature of the intervention*—*it might possibly be relatively easier to refuse outcome evaluation when the only contact with study-related staff has been at assessment and subsequent ‘contact’ is only text messages, unlike actual human contact in a face to face counselling intervention. Strategies such as check-in calls by the research team at regular intervals could potentially reduce loss to follow up at outcome evaluation.

This iterative modelling and feasibility testing process that we followed is critical to the intervention development process, allowing for revisions to the intervention to overcome engagement barriers, through feedback loops. These feedback loops allowed us to revise the intervention based on data we collected and to then test the revised intervention for acceptability and feasibility. Some key findings during the intervention delivery that allowed us to revise the intervention are described in the results above.

The quantitative outcome evaluation indicated a favorable change in the patterns of drinking. Although this was either statistically significant and/or in the desired direction, the absence of a counterfactual in the form of a control arm and the small sample sizes, precludes making any conclusion about the role of the intervention in this change or the precision of the findings respectively. However, in combination with the change in drinking patterns reported in the qualitative study, there is a strong rationale to further testing of the intervention for effectiveness using an appropriate study design. Finally, the small sample size has also resulted in wide 95% confidence intervals which reflect the limited precision of the findings.

This study demonstrated the importance of such preliminary testing while developing an intervention. It allowed us to make changes to the intervention content as well as delivery to enhance its acceptability and feasibility, gave us preliminary indication of its potential impact on drinking outcomes. Some findings reinforced the acceptability and feasibility of the existing content, delivery modes, and potential mechanisms of change, while other findings conflicted with the preliminary intervention as designed through formative research. The latter further emphasized the need for feasibility and acceptability testing to complement formative research based on the existing evidence base and perceptions of experts and intended recipients on what might be suitable for a particular context. After establishing the feasibility of the intervention we conducted preliminary testing in a pilot RCT [32] which, among other things, allowed us to establish the sample size that will be needed for a definitive RCT to test the effectiveness of the intervention. We also conducted an exploratory analysis to examine if there was any relationship between AUD and stress (PSS) or activity levels (IPAQ) but did not find any significant associations (Appendix S3).

The strengths of our study lie in its participatory nature and the triangulation of quantitative and qualitative findings. Some of the limitations around study design and sample size have already been addressed above and include high dropout rate and limits to generalizability resulting from the limited participation of women. An additional limitation which affects the interpretation is that every participant did not receive the same intervention, because it was revised during the study in response to the feedback loops. However, this very limitation can also be viewed as a strength of an intervention development study as it allows for adequate testing of such iterative revisions to the intervention.

The outcome of our study is a mobile phone delivered BI for hazardous drinking that has been suitably adapted to ensure feasibility and acceptability for delivery in the Indian context. This is the first such intervention for hazardous drinking in India or any other LMIC, as far as we are aware. If found to be cost-effective in a definitive RCT, our intervention has the potential to address the several barriers to implementation of BIs in low resource settings.

## Supplementary Material

Supplementary_material_FINAL_oqae045

## Data Availability

The data underlying this article will be shared on reasonable request to the corresponding author.
