# Supplementary material for: The acceptability and feasibility of a mobile phone delivered brief intervention for hazardous drinking in India
Source: Oxf Open Digit Health. 2024 Oct 29;2:oqae045. doi: 10.1093/oodh/oqae045 (PMC11932400; doi:10.1093/oodh/oqae045)
Supplement: Supplementary_material_FINAL_oqae045 [file Supplementary_material_FINAL_oqae045.docx]

**SUPPLEMENTARY MATERIAL**

**The Acceptability and Feasibility of a Mobile Phone Delivered Brief Intervention for Hazardous Drinking in India**

Abhijit Nadkarni^a,b^*, Danielle Fernandes^b^*, Richard Velleman^b,c^, Anastasia Onyango^b,d^, Seema Sambari^b^, Ethel D’Souza^b^

*Joint first author

^a^Centre for Global Mental Health, Department of Population Health, London School of Hygiene and Tropical Medicine, UK

^b^Addictions and Related Research Group, Sangath, Porvorim, Goa, India

^c^Department of Psychology, University of Bath, Bath, UK

^d^Department of Scoiology, Harvard University, Boston, USA

Corresponding author- Abhijit Nadkarni, Centre for Global Mental Health, Department of Population Health, London School of Hygiene and Tropical Medicine, London UK WC1E 7HT

[abhijit.nadkarni@lshtm.ac.uk](mailto:abhijit.nadkarni@lshtm.ac.uk)

**Appendix 1: AMBIT intervention content**

| **Intervention component** | **Description** |
| --- | --- |
| Safe drinking | Specific safe drinking tips |
| Self-awareness | How to assess personal alcohol use habits, acknowledge problem drinking, and increase self-motivation to change |
| Alcohol reduction | Encourage reduction alcohol intake |
| Goal-setting | Identify and modify progression towards goals for making changes to drinking behaviour |
| Situational content | Relatable information about common situations involving alcohol consumption and tips to respond to them in a healthy manner |
| Self-reflection | Prompts to observe, assess, and analyse drinking behaviour, and decide next steps |
| Drinking management | Prevention, risk reduction, and coping methods within the context of hazardous drinking |
| Risk management | How to avoid and strategize for specific risky drinking situations that may act as barriers to achieving drinking goals |
| Motivation | Incentivise and encourage healthy behaviour adoption and maintenance |
| Drinking alternatives | Alternatives to drinking that can help reduce desire to consume alcohol |
| Review | Series of questions to assess the user's drinking behaviour in previous week |
| Information about other resources for help | Information about relevant health resources |
| Urge management | Skills to overcome drinking urges |
| Actionable feedback | Information to influence future health behaviour |
| Maintenance and relapse prevention | Assignments to help practise and maintain goals for healthier drinking behaviours |
| Goal management | Support setting and maintaining goals and provide rewards for goal achievement |
| Check- in messages | Check -in messages to ensure movement towards the desired drinking goal |

**Appendix 2:** **Difference in drinking patterns at baseline and follow up.**

| **Outcome** |  | **All participants (n=16)** | |
| --- | --- | --- | --- |
|  |  | **Median (Range)** | **p** |
| Percent Days Abstinent (PDA) | Pre-treatment  Post treatment | 50.0 (0.0-92.9)  89.3 (0.0-100.0) | 0.0009 |
| Percent Days Heavy Drinking (PDHD) | Pre-treatment  Post treatment | 3.6 (0.0-57.1)  0.0 (0.0-100.0) | 0.66 |
| Standard drinks | Pre-treatment  Post treatment | 9.7 (0.0-65.5)  3.6 (0.0-250.9) | 0.30 |

**Appendix 3: Relationship between alcohol use disorder, stress and activity**

|  | **AUDIT score <8** | **AUDIT score >8** | **p** |
| --- | --- | --- | --- |
| **IPAQ** | **N=60** | **N=30** | 0.95 |
| Low physical activity | 19 (31.7) | 9 (30.0) |  |
| Moderate physical activity | 23 (38.3) | 11 (36.7) |  |
| High physical activity | 18 (30.0) | 10 (33.3) |  |
| **PSS** | **N=89** | **N=36** | 0.43 |
| Low perceived stress | 24 (27.0) | 6 (16.7) |  |
| Moderate perceived stress | 59 (66.3) | 28 (77.8) |  |
| High perceived stress | 6 (6.7) | 2 (5.6) |  |
